# True Legacy

**DOI:** 10.1055/a-2836-2196

**Published:** 2026-06-15

**Authors:** Joon Pio Hong

**Affiliations:** 1 Department of Plastic Surgery, Asan Medical Center, University of Ulsan, Seoul, South Korea

**Figure:**
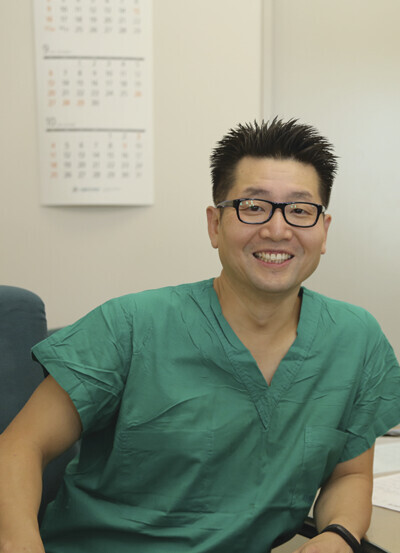
Joon Pio Hong, MD, PhD, MMM (Editor Emeritus of APS)

The concept of “legacy” is frequently invoked when reflecting on the achievements of a career, yet its meaning has evolved significantly over time. Contemporary definitions describe legacy as the enduring impact of past actions, events, or intentions, particularly those associated with an individual's life. Etymologically derived from the Latin *legatus* —meaning ambassador, envoy, or deputy—and *legare* , “to send with a commission,” the term originally referred to a person entrusted with authority. By the late medieval period, its meaning shifted toward property or material inheritance left after death. In the modern era, the definition has expanded to encompass intangible dimensions such as reputation, values, and lasting historical influence. Thus, legacy today represents not only what is left behind materially, but also the enduring imprint one's actions leave on individuals, institutions, and society.

As we age, we often become increasingly preoccupied with legacy because it speaks to a fundamental human concern: The desire for meaning beyond one's own lifetime. Yet unless one has created a truly transformative inflection point in history, few names endure across generations. When I ask my residents about plastic surgeons who once transformed the field—such as the late McCarthy, Converse, O'Brien, and others—they rarely recognize their names or recall their contributions. And yet, many of us aspire to leave contributions that will improve our field, perhaps even redefine it—sometimes at the expense of others. Even those considered giants of their era may be scarcely remembered two generations later.

This raises an important question: What, then, is the true meaning of legacy, and how should we think about it as we live our lives today? If legacy means leaving an enduring impact through our actions, events, or intentions, then for most of us, that impact will not be global or eternal. For ordinary individuals, legacy lies in the lives we touch directly—our family, friends, trainees, colleagues, and patients. While a select few may be remembered by history, most of us will live on primarily in the memories of those with whom we shared meaningful relationships.

When our loved ones and colleagues remember us, speak of us, and carry forward something of what we taught or embodied, that is legacy. The more important question, therefore, is not whether history will remember our names, but how we choose to live today so that our presence leaves a lasting and positive imprint on those around us.

Do not waste your time competing, harboring jealousy, or attempting to discredit one another. Instead, focus your energy on being candid, loving, and genuinely invested in one another's success. Celebrate together. Grieve together. Above all, be patient with one another. Be an ambassador of generosity—share your feelings openly and teach what you have learned with your heart. When I see former trainees or colleagues surpass what I have contributed, I see the continuation of a legacy I was fortunate to help shape. We live only a short life. Do not focus too much on what legacy you want to leave, but rather on how you want to be remembered by the people around you—that is true legacy.

